# Important Legal Decision in Medical Consultations

**Published:** 1899-12

**Authors:** 


### Important Legal Decision in Medical Consultations.

The *Boston Medical and Surgical Journal* for October 26 says that a case of considerable interest to medical men has recently been ordered to be retried by the appellate term of the Supreme Court. A bill of two homeopathic physicians, of seventy dollars for the attendant, and a hundred and seventy-five dollars for the consultant (who had made six visits), in a case of fractured elbow, was disputed by the patient, who was left with a stiff joint, and interposed with a counter claim for five hundred dollars damages for malpractice. In the sixth municipal court the full amount of the bill was awarded to the physicians. In giving his opinion in favor of reversal, Justice McLean of the Supreme Court, concluded as follows:

"There is no justification, by custom or otherwise, in plaintiff's employment of Dr. Roberts (the consultant) without a frank and full statement of the situation to the patient and the defendant (the patient's husband), and learning their wishes concerning the professional persons to be brought in. There can not be properly applied to the facts shown here any custom multiplying ordinary professional charges five or ten times under the shield of a layman's ignorance, because it is subversive of justice that charges should be so largely increased by a custom not made known at all to the patient or to her husband."

